# Experimental Verification of a Predicted Intronic MicroRNA in Human NGFR Gene with a Potential Pro-Apoptotic Function

**DOI:** 10.1371/journal.pone.0035561

**Published:** 2012-04-27

**Authors:** Sepideh Parsi, Bahram M. Soltani, Ebrahim Hosseini, Samaneh E. Tousi, Seyed J. Mowla

**Affiliations:** Molecular Genetics Department, Faculty of Biological Sciences, Tarbiat Modares University, Tehran, Iran; University of Cordoba, Spain

## Abstract

Neurotrophins (NTs) are a family of secreted growth factor proteins primarily involved in the regulation of survival and appropriate development of neural cells, functioning by binding to their specific (TrkA, TtkB, and TrkC) and/or common NGFR receptor. NGFR is the common receptor of NTs, binding with low-affinity to all members of the family. Among different functions assigned to NGFR, it is also involved in apoptosis induction and tumorigenesis processes. Interestingly, some of the functions of NGFR appear to be ligand-independent, suggesting a probable involvement of non-coding RNA residing within the sequence of the gene. Here, we are reporting the existence of a conserved putative microRNA, named Hsa-mir-6165 [EBI accession#: FR873488]. Transfection of a DNA segment corresponding to the pre-mir-6165 sequence in Hela cell line caused the generation of mature exogenous mir-6165 (a ∼200,000 fold overexpression). Furthermore, using specific primers, we succeeded to detect the endogenous expression of mir-6165 in several glioma cell lines and glioma primary tumors known to express NGFR. Similar to the pro-apoptotic role of NGFR in some cell types, overexpression of pre-mir-6165 in U87 cell line resulted in an elevated rate of apoptosis. Moreover, coordinated with the increased level of mir-6165 in the transfected U87 cell line, two of its predicted target genes (Pkd1 and DAGLA) were significantly down-regulated. The latter findings suggest that some of the previously attributed functions of NGFR could be explained indirectly by co-transcription of mir-6165 in the cells.

## Introduction

Nerve growth factor receptor (NGFR) (NC_000017.10) is a multi-functional cell surface receptor in many human cell types including some adult brain cells. This gene can induce apoptosis and is also involved in injury, nervous system development and regeneration [1]. NGFR expression is induced in many pathological conditions, such as atherosclerosis [2], ischemia [3], diabetes [4]–[5] and cancer [6]–[14]. NGFR acts as a tumor suppressor in most cases of cancers, causing apoptosis and suppression of metastatic invasion. Contrary, NGFR can induce invasion and metastasis in glioma [15] and melanoma [9].

There are some transcription factors which regulate NGFR gene expression in conditions like hypo-osmolar stress and injuries [16]. There are also an increasing list of ligands, co-receptors and adaptor proteins which interact with NGFR, involving this gene in different signaling pathways ending to cell death and/or survival [17]. Recent publications suggest a ligand independent activation and also a non-NGF ligand activation of NGFR signaling [18]–[19]. It remains to be found if non-coding RNAs such as miRNAs are involved in this regulation as well.

miRNAs are endogenous small non-coding RNAs about 21–23nt, each capable of interfering with dozens of target mRNAs through complete or partial complementarities. The miRNA genes are transcribed by RNA polymerase II or III and the primary transcript termed pri-miRNA, is quickly trimmed into ∼70nt long pre-miRNA precursor. The pre-miRNAs are then transported to the cytoplasm and further processed to functional miRNAs, which exert their regulatory functions by complementary binding to their target genes [20]. To date, more than 1000 human miRNAs have been published in mirbase database [21], among which 40% are located within introns of protein-coding ‘host’ genes [22]–[27] and appear to be conserved across the species.

Identification of novel miRNA by forward genetics has been tedious as a result of the small sizes of miRNAs as well as their tolerance to mutations that do not affect their seed sequences [28]. On the other hand, the computational prediction of non-coding RNAs (ncRNAs) has proven to be fast, cheap and effective [29]–[30]. After prediction of a miRNA gene, experimental verification is necessary to demonstrate its exact mature sequence and function. Briefly, some major characteristics such as the hairpin-shaped stem loop structure, high evolutionary conservation and high minimal folding free energy are important features used in the computational identification of novel miRNAs [31]. Here bioinformatics tools were used to search for hairpin structures within the NGFR intronic and exonic regions. One of the NGFR resided hairpin structures showed all the bioinformatics characters of producing a real miRNA. Later, accumulative experimental evidences for the first time showed the presence and the functionality of this novel NGFR intronic miRNA in human cells.

**Table 1:** Primers and oligos used in this research.

Gene and primer name	Sequence 5′ to 3′
NGFR	Forward: CCGAGGCACCACCGACAACC Reverse: GGGCGTCTGGTTCACTGGCC
U48	Forward: TGACCCCAGGTAACTCTGAGTGTGT
Precursor	Forward: CAGCAGGTCAGCAGGAGGTGAGGGG Reverse: GGGAGGGGCTGGAGCCAGGACAGG
Putative mir1 (Pm1)	CAGCAGGAGGTGAGGGGAG
Putative mir1^*^ (Pm1^*^)	TGTCCTGTCCTGTCCTCTCCTG
Anchored Oligo dT	GCGTCGACTAGTACAACTCAAGGTTCTTCCAGTCACGACG (T)17N
Universal outer primers	(a) AACTCAAGGTTCTTCCAGTCACG(b) GCGTCGACTAGTACAACTCAAG
Pkd1	Forward: GTTCTCAGGCCTCCACGCTGAG Reverse: AGGGCCAGCACACCAGACTCTTAGA
DAGLA	Forward: ACTGGCCTTGCCCTGGAGCT Reverse: CGCAACCACTGGCGACAGCA

**Figure 1:**
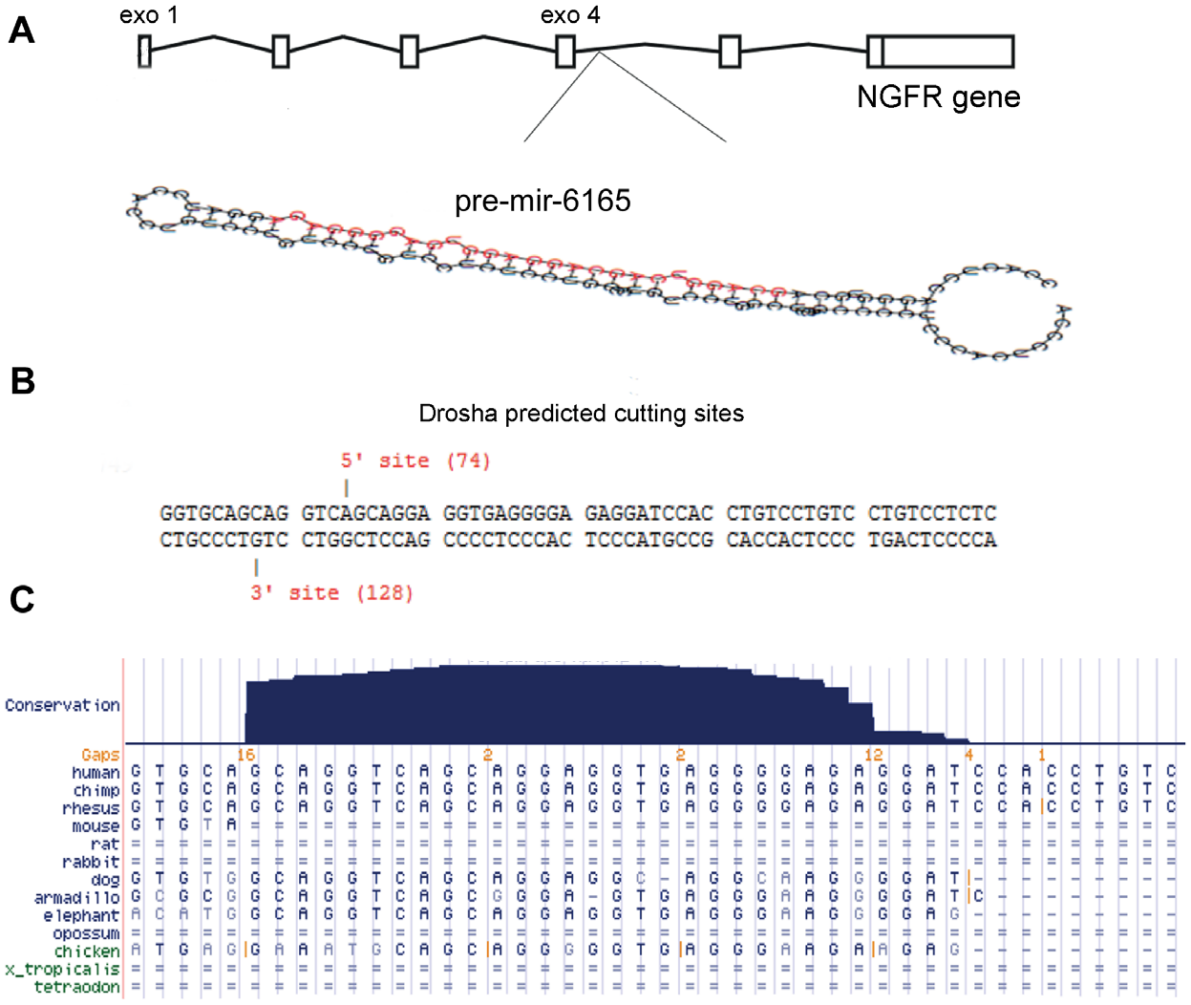
Prediction of pre-mir-6165 within the 4^th^ intron of human NGFR gene. A) Position of predicted hairpin structure within the human NGFR gene is shown in the 4^th^ intorn. This hairpin is predicted to produce Hsa-mir-6165 which is shown as red colored sequence on the stem loop. B) Prediction of Drosha enzyme 5' and 3' cutting sites on the sequence of stem loop by Microprocessor SVM. C) Blat search result shows a strong conservation of Hsa-mir-6165 between human, rhesus, dog and elephant.

## Materials and Methods

### Bioinformatics Tools and Studies

To search for the possible hairpin structures within the area of interest, SCC profiler [32] and miPRED [33] online classifier [http://www.bioinf.seu.edu.cn/miRNA] programs were employed. For the identification of putative miRNA precursors in NGFR introns CID-miRNA [34] was used along with the prediction of Dorsha processing sites using Microprocessor SVM (https://demo1.interagon.com/miRNA/) program [35]. Mireval [36] (http://tagc.univ-mrs.fr/mireval/) and mirbase (http://www.mirbase.org/index.shtml) databases were also used to determine the degree of conservation of mir-6165 and its precursor sequence along with blat [37] search against human genome and other organisms.

DIANA-microT web server [38]–[39] (http://diana.cslab.ece.ntua.gr/pathways/) was employed in order to find potential target genes for this novel miRNA. The hsa-mir-6165 prediction was also performed by using MatureBayes [40], pmirp [41] and mirz [42] online tools. To search for a putative promoter sequence upstream of (pri-mir-6165) gene, Promoter 2.0 Prediction Server (http://www.cbs.dtu.dk/services/Promoter/) was employed [43]. Diana-mirpath [44] and geneset2miRNA [45] online tools used to find the pathways in which mir-6165 is involved. To search for the co-expression of miRNA target genes with miRNA host gene, GENEMANIA [46] online tool was used. Gene ontology analysis of mir-6165 potential targets was done by using Gene Ontology Functional Analysis Tool (DAVID) [47].

**Figure 2:**
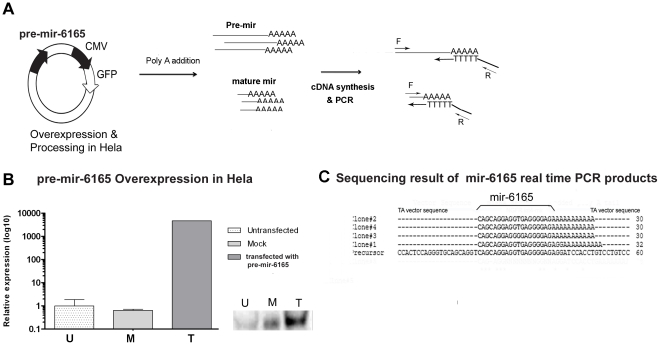
pre-mir-6165 overexpression in the Hela cells and detection of Hsa-mir-6165 mature form. A) Schematic presentation of pre-mir-6165 cloning, overexpression and its RNA adenylation followed by cDNA synthesis, using a universal anchored-oligo-dT primer. For the amplification of precursor, first strand cDNA was PCR amplified using precursor specific F-primer and reverse anchor primer on the oligo-dT tail. For the amplification of mature miRNA, predicted mir-6165 sequence was used as the forward primer. B) Hsa-mir-6165 increased production (200,000x) following transfection of Hela cells with its precursor. In the untransfected cells (U) or scrambled control (M), the level of this miRNA was lower compared to the transfected cells (T). C) Four sequencing result of TA vector clones containing mir-6165 real time PCR products, are compared to the precursor sequence. The sequences between the laboratory added polyA and the upstream vector sequence are considered as mature miRNA. Sequencing of clones #2, #3 and #4 shows that prediction of Hsa-mir-6165 sequence has been correct. Clone#1 also shows the similar sequence plus AGG extra nucleotides which is considered an iso-mir for the miRNA.

### Cell Culture

Hela and U87MG (both obtained from Pasteur Institute, Iran) cell lines were cultured in RPMI-1640 media (Invitrogen), supplemented with 10% fetal bovine serum (FBS) (Invitrogen), 100 U/ml penicillin and 100 µg/ml streptomycin (Sigma), and incubated in 37°C with 5% CO2. NT2 cells [48] were cultured in DMEM-HG containing 10% heat-inactivated FBS, 100 U/ml penicillin and 100 ug/ml streptomycin.

### Tissue Samples

Fresh surgical tissue biopsies of meningioma, glial, astrocytoma and oligodendroglioma were kindly provided by Imam Hospital [49].The samples were stored and processed as previously described in the same reference.

### RNA Extraction

Total RNA was extracted from cell lines using Trizol reagent according to the manufacturer's protocol (Invitrogen). Residual DNA was removed using RNAase-free DNAase I (Takara) at 37°C for 30 min followed by heat inactivation at 65°C for 10 min by addition of EDTA.

Real-time PCR detection of precursor and mir-6165 mature form.

According to the predicted precursor and mature miRNA sequences, primers were designed for quantitative PCR (Table 1), using NCBI primer-blast, MWG Operon online PCR primer design tool (www.eurofinsdna.com) and primer bank (http://pga.mgh.harvard.edu/primerbank/). Briefly, 1 ug of total RNA sample was used in 20 ul Poly A tailing reaction containing; 2.5 U PolyA polymerase (BioLab), 2 ul of 10 mMol ATP and incubated at 37°C for 10 min. Then, the total poly adenylated RNA was used in 10 µL first strand cDNA synthesis reaction using PrimeScript II reverse transcriptase (Takara) and a cocktail of specific oligo-dT primers containing a universal anchor (Table-1). cDNA synthesis reaction was performed at 42°C for 30 min and terminated at 80°C for 5 s. Real-time quantitative PCR was performed using standard protocols on an ABI PRISM 7500 instrument (Applied Biosystems). Briefly, the run method profile consisted of: stage 1, 95°C for 5 s,stage 2, 60°C for 20 s; stage 3, 72°C for 34 s. Stage 2 was repeated for 45 cycles. Continuous melt curve stages included a first step of 95°C/15 s, step 2 at 60.0°C for 1 min, 95°C/30 s and a last step of 60°C for 15 s. Total PCR products were cloned in TA vector (Fermentas) and were sequenced (Genfanavaran Co.).

### Overexpression of mir-6165 Precursor in Hela Cell Line

Human genomic DNA was extracted from white blood cells using standard protocol [50]. 84 bp fragment representing the predicted pre-miRNA was PCR amplified, using precursor forward and reverse primers (Table 1), using PFU polymerase (Takara) and cloned in modified pEGFP-C1 expression vector with two CMV promoters. The 84 bp fragment was cloned in the vector using Not1 and EcoRV restriction enzymes. Recombinant vectors were propagated by miniprep (Qiagene Co.) and 2 ug of this DNA was used for lipofectamin (Invitrogen) transfection of Hela and U87 cell lines in 24 well plates containing about 2×10∧5 cells per well. GFP expression was visualized by a florescence microscope (Nikon eclipse Te2000-s).

### Cell Cycle Analysis

After 34 hours, the U87 and Hela cells over expressing pre-mir-6165 were harvested and stained with propidium iodide (PI) and Annexin V (Roche) according to the manufacturer’s protocol. All samples were analyzed with a FACS Calibur flow cytometer with Cell Quest software (BD Biosciences). All assays were carried out in duplicates.

### Statistical Analysis

Real time experiments were run in duplicates. Real time data were analyzed using ΔΔCT method by DataAssist software V3.0 and normalized by endogenous control U48 small nucleolar RNA gene (SNORD48) and B2m or globalization method [51]. Other statistical analysis was performed with GraphPad Prism 5.04 (GraphPad, San Diego, CA). For apoptosis studies, data showing percent of early apoptotic cell population within negative group and test group, compared with each other by Repeated Measures ANOVA test, and followed by Bonferroni test using GraphPad. Data were considered statistically significant, when P-values were <0.05.

**Figure 3:**
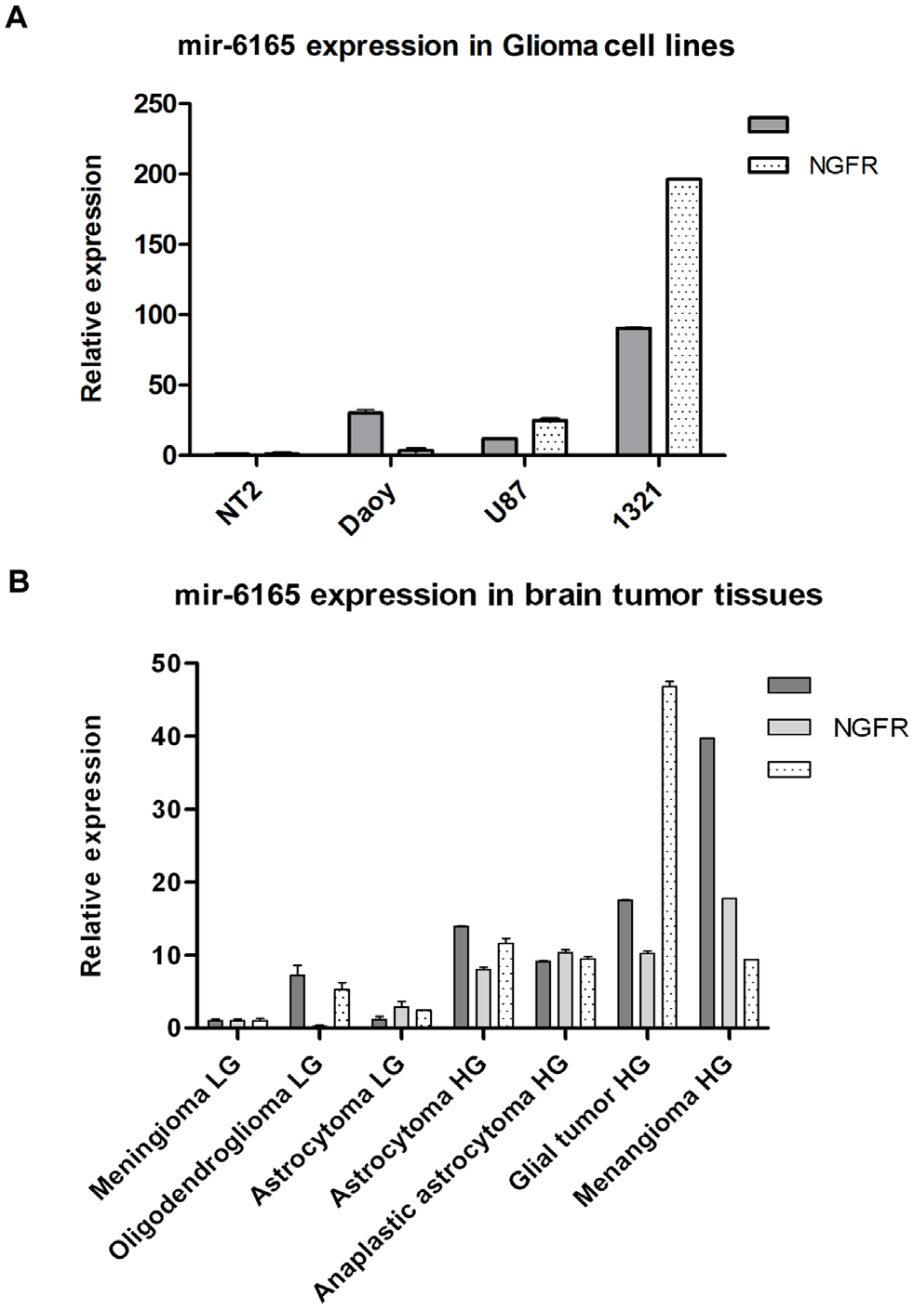
Detection of Hsa-mir-6165 in the brain derived cell lines and biopsies. A) NGFR and mir-6165expression profile in some glioma cell lines is compared to non-glioma NT2 cell line Daoy, 1321N1, U87 (glioma cell lines) and NT2 (non glioma cell line) were used for detection of Hsa-mir-6165 expression. U48 small neucleolar RNA was used as internal control for the amplifications. In glioma cell lines Hsa-mir-6165 expression level was higher than NT2 cell line. B) Relative, Hsa-mir-6165 and its precursor expression levels in various human glioma tissue samples. The expression level of Hsa-mir-6165 in the tumor samples were compared to the lowest grade of tumors. U48 small nucleolar RNA gene (SNORD48) was used for normalizing the expression levels. Error bars indicate standard deviation (SD) of duplicate experiments. Pearson’s test confirmed a positive correlation between NGFR and its intronic miRNA (p = 0.0065). In all of the high grades (HG) tissue samples, the level of NGFR and mir-6165 were higher than the low grad (LG) samples.

**Figure 4:**
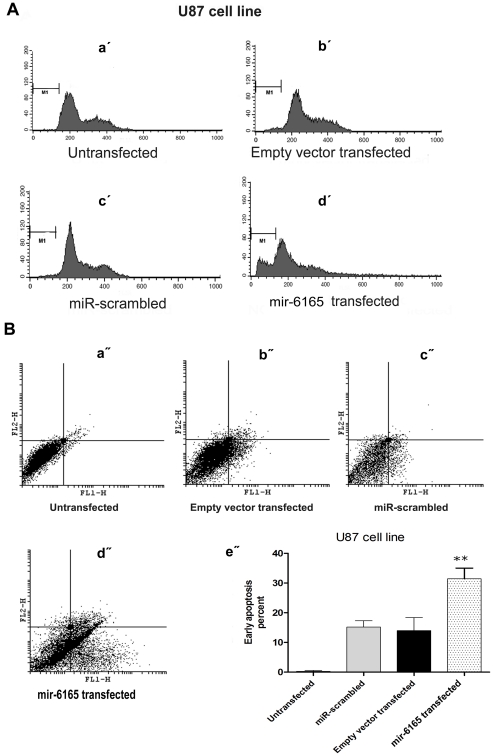
mir-6165 overexpression in U87 cell line induces apoptosis. A) PI staining of U87 cells 34 hours post transfection was done to investigate the effect of mir-6165 on cell cycle. A dramatic change was observable toward sub-G1 stage in the cells overexpressing mir-6165 compared to negative controls (a′- d′).B) Annexin-PI staining of the U87 cells overexpressing mir-6165 shows, the most of the cells have entered early apoptosis stage compared to negative control and the result is consistent with PI staining in the previous section (a″- d″). The gate setting distinguished between living (bottom left), necrotic (top left), early apoptotic (bottom right), and late apoptotic (top right) cells. Repeated Measures ANOVA analysis shows that the changes observed in flow cytometry of U87 cells is extremely significant (p<0.05) between negative controls and the cells overexpressing mir-6165 (e″).

## Results and Discussion

### Prediction of a Novel Intronic miRNA Within the Human NGFR Gene

Due to their implications in several diseases including cancer, miRNAs are under intensive research aiming at novel pharmacological interventions. Computational tools have been used for efficient prediction of novel miRNAs and their target genes [30]. After several failed PCR attempts to amplify the area spanning the 4^th^ intron of rat NGFR gene, chr10: 84267661–84265746[-] UCSC nov.2004 (Baylor 34 rn4), we used mFOLD program (http://mfold.rna.albany.edu) to search for possible hairpin structures in this region. This program introduced multiple hairpin structures in the 4^th^ intron of NGFR genes both in human and rat genomes. Using miRNA prediction HMM based tool (SCC profiler), several potential miRNA precursors were identified, however, only one of them, named pre-mir-6165, showed the criteria of producing a real human intronic miRNA locating; hg19, chr17: 47588166 – 47588269 (Figure 1A). Microprocessor SVM program also predicted a Dorsha processing site for pre-mir-6165 (Figure 1B). To date for mir-6165, no identical miRNA has been reported in the mirbase database, except a weak resemblance to mir-328a in rat and mouse. Furthermore, Mireval online tool identified this novel precursor with strong conservation and with no homology to other miRNAs. Using blast search, it was demonstrated that both sequence and structure of pre-mir-6165 was conserved in mammals, while mir-6165 was mostly conserved in primates (Figure 1C). Overall, accumulated bioinformatics evidences suggested the existence of a novel microRNA. Firstly, CIDMIR, Pmirp, MatureBayes, miR-abela -MirZ and Microprocessor SVM softwares all recognized pre-mir-6165 with significant scores. These tools consider conservation, expression level and other characters in order to predict the possibility of a miRNA production. Secondly, precursor sequence as a query produced 9 hits with 100% identity at the level of 20–27 bp in the Blat search against human genome. Four of these yet uncharacterized hits show common characters of mir-precursors and are very similar to the already reported miRNA precursors in the mirbase database. Others have used similar method for the discovery of new miRNAs [32]. Thirdly, conservation of seed [52] as well as the rest of mir-6165 sequence in human genome is a strong bioinformatics supporting evidence for the presence of this miRNA (Figure 1C). This miRNA is not clustered and is weakly conserved between taxa, the same property is already reported for certain miRNAs which are not conserved [53], not clustered [54], have temporal and cell-specific expression patterns [55], and have no homology to other miRNAs [35].

**Figure 5:**
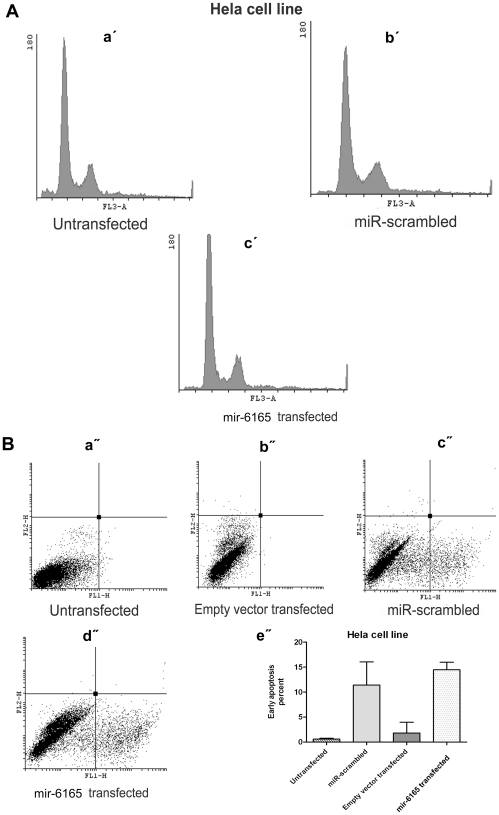
mir-6165 overexpression in Hela cell line. A) PI staining of Hela cells overexpressing Hsa-mir-6165 did not show any significant change in the stages of cell cycle after 34 hours post transfection (a′- c′). B) Annexin-PI staining of the Hela cells shown in figures a″- d″. Repeated Measures ANOVA analysis shows that the changes observed in annexin test of Hela cells were not significant between negative controls (scramble) and the test group (e″).

### Overexpressed pre-mir-6165 is Efficiently Processed in the Hela Cells

According to the Oncomine [56], the database for the expression profile of the genes, NGFR gene expression (and as a result pre-mir-6165) is very low in Hela cell line. In an attempt to overexpress pre-mir-6165 in Hela cells and check for the production of mature mir-6165, a corresponding 84 bp DNA fragment was amplified by PCR from human genomic DNA and ligated to the pEGFP-C1 modified expression vector under CMV promoter (Figure 2A). The same vector without insert and/or containing scramble DNA, were used as a negative controls in further experiments. Transfection rate of these cells was monitored via fluorescent emission of GFP, and the best transfected culture was used for RNA extraction and gene expression studies. In Hela cells which were transfected with the pre-mir-6165 construct the level of mature mir-6165 elevated by a factor of 200,000 fold. The result indicates that mir-6165 precursor cloned under CMV promoter is efficiently expressed and processed to a mature form (Figure 2B). Mature mir-6165 real-time PCR products showed an expected size on the acrylamid gel and were subsequently cloned in the TA vector for sequencing. The sequencing results of three independent clones showed the exact sequence of expected mir-6165 before polyA tail, indicating correct prediction of 3′-end of the miRNA (Figure 2C). On the other hand, sequencing result from one of the TA clones showed 2 bp extra nucleotides after the predicted 3′ end for mir-6165. This might be the result of subtle variation in the RNA ends corresponding to Drosha and Dicer cleavage sites.

**Table 2:** Top ten predicted targets for novel Hsa-mir-6165 according to DIANAmicro T v.3.

Rank	Gene name	Ensembl Gene Id	miTGscore	Precision	SNR
1	DAGLA	ENSG00000134780	19.21	0.82	3.97
2	PKD1	ENSG00000008710	18.00	0.82	3.97
3		ENSG00000196518	18.00	0.82	3.97
4	LRRC27	ENSG00000148814	18.00	0.82	3.97
5	KIAA0258	ENSG00000107185	16.09	0.78	3.97
6	VANGL2	ENSG00000162738	16.00	0.78	3.97
7	RHPN1	ENSG00000158106	16.00	0.78	3.97
8	WDTC1	ENSG00000142784	15.00	0.78	3.97
9		ENSG00000138944	15.00	0.78	3.97
10	MECP2	ENSG00000169057	15.00	0.78	3.97

The Signal to noise ratio (SNR) is calculated by the DIANA-microT algorithm and is based on a comparative analysis of the real miRNA versus a set of mock miRNAs. Higher miTG scores correspond to higher possibility of correct prediction. Greater values of SNR correspond to better distinction from the mock background [38].

**Figure 6:**
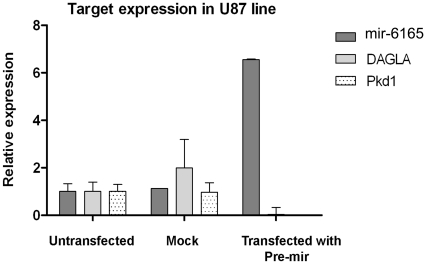
Down regulation of hsa-mir-6165 target genes following its precursor overexpression. Down regulation of DAGLA and Pkd1 predicted target genes following the overexpression of Hsa-mir-6165 compared to the scrambled negative controls. Data of expression were globally normalized against U48, U6 and B2m as endogenous controls.

The minimum size of these miRNAs was submitted to EBI data base under the extension number of; [EBI accession#: FR873488, FR873489]. Overall, these experiments demonstrate that Hela cells are able to process the predicted pre-mir-6165 to its predicted mature miRNA form.

### Experimental Detection of Endogenous mir-6165 and its Precursor in Glioma Cell Lines and Brain Tumors

Promoter 2.0 Prediction Server did not predict an independent promoter for mir-6165, therefore, its transcription is supposed to be through NGFR host gene promoter. NGFR is expressed shortly in few normal cell lineages during the development, but it is expressed at a detectable level in the human Glioblastoma U87-MG cell line [15], [56]. For this reason, U87-MG and other glioblastoma cell lines were chosen for the experimental detection of mir-6165 and its precursor. The fact that one or both of mir and miRNA-star sequence (miRNA*), the complementary strands of functional mature miRNA, might exert function, two primers were designed; one exactly identical to the predicted mir-6165 (called Pm1 primer, Table-1) and the second primer was identical to its complementary mir* (called Pm1* primer). After Poly-A addition to the total RNA extracted from several glioblastoma cell lines, cDNA was synthesized using anchored oligodT primer (Table-1). Later along with NGFR gene, the mature form of mir-6165 was amplified from RNA samples of Daoy, 1321N1, U-87 and NT2 cell lines, using Pm1 primer (Figure 3A). In this figure, mir-6165 expression in Daoy, 1321N1, U87 (brain tumor-derived) cell lines are compared to a non-glioma (a human teratocarcinoma, NT2) cell line. mir-6165 and its host gene (NGFR) showed higher expression level in the brain tumor-derived cell lines, compared to the NT2 cells. This data supports the correct prediction of mir-6165 at the first place and also points to the origin of the cells (glioma) which express this miRNA at a detectable level.

Further supporting evidences were provided through the detection of endogenous precursor and mature form of mir-6165 in the brain tumor biopsies (Figure 3B). mir-6165 endogenous expression was detected using real-time PCR on seven brain tumor tissue samples. Expression data was normalized using U48 endogenous control, and using one of the lowest degree meningioma samples as a reference. Along with the clear detection of endogenous mir-6165 in these tissue samples, a strong correlation was observed between mir-6165 and NGFR gene expression, which was further confirmed with Pearson’s test (p = 0.0065). In all of the high grade tissue samples, the level of NGFR and mir-6165 were higher than the low grade samples (Figure 3B).

In a neurotrophin-dependent manner, NGFR has been suggested as an important regulator of glioma invasion [15]. In that report, NGFR-positive cells within the glioma tumor samples are more migratory than the NGFR-negative glioma cells. Consistent with that report, we have seen upregulation of NGFR in higher grades of brain tumors.

A possible molecular mechanism in which mir-6165 co-operates with NGFR toward glioma invasion remains unknown; however, analysis of putative targets of mir-6165 by DAVID software [47] showed >50% of the predicted targets genes are those expressed in brain related tissues (Figure S1) among them Pkd1 and DAGLA genes were down regulated in tested tumor samples (data not shown).

### Cell Death Effect of pre-mir-6165 Overexpression in U87 Cell Line

In order to examine the effect of pre-mir-6165 overexpression on the rate of apoptosis induction in the transfected cells, flow cytometry was performed using propidium iodide (PI) and annexin. This method has been reliably used to show the involvement of miR-16, let-7a and miR-34a in apoptosis [57]. Compared to the mock transfected control cells, no statistically significant apoptosis induction was detected 34 hours post transfection in Hela cells using PI staining. However, transfected U87 cells showed more than 10% elevation in sub-G1 cell population (Compare figure 4A with 5A). P value for such increased cell death rate was calculated by Repeated Measures ANOVA analysis and the results were highly significant (p = 0.0009). Annexin test shows the early apoptosis rate of the transfected cells and is more sensitive than PI test. This test showed ∼40% of the transfected U87 cells were in early apoptosis (p = 0.003) (Figure 4B). Overall, our results suggest a pro-apoptotic role for mir-6165 in the U87 cells, but not in the Hela cell line (Figure 5).

Lack of significant apoptotic effect of mir-6165 in transfected Hela cells may be due to the cellular environment or context of the cell [58] or lack of its partner(s) or target(s) in this cell line.

Accumulative experimental evidences from the detection of endogenous mature mir-6165 in the brain tumor samples to the detection of exogenous mir-6165 in the transfected Hela cells and its overexpression effect on the cell death rate, all emphasis the functionality of this miRNA.

### Down Regulation of Predicted mir-6165 Target Genes upon its Overexpression

The sequence of mir-6165 was used as query for target prediction in DIANA-microT online tool and its top ten candidate genes are listed in Table 2. In the glioblastoma cell lines in which NGFR and mir-6165 are expressed, some of these target genes are down regulated based on the information from Oncomine database. DAGLA gene (Neural stem cell-derived dendrite regulator) is the highest scored target gene of the list and its miRNA recognition element (MRE) is highly conserved among other organisms. The interaction between this target and mir-6165 was further analyzed using RNAHybride (http://bibiserv.techfak.uni-bielefeld.de) online tool. Strong complementation was observed between the mir-6165 and its target MRE element in the DAGLA gene.

For experimental verification of the mir-6165 target genes, real-time specific primers were designed for (DAGLA and PKD) predicted target genes. Overexpression of pre-mir-6165 in Hela cells ended in 200,000 fold increase of mature form, 34h post transfection but DAGLA and PKD expression level still remained undetectable. In contrast to Hela cells, DAGLA and PKD genes were expressed at a detectable level in the untransfected U87 cells. Upon overexpression of mir-6165 in the U87 cells, a significant down regulation of these target genes was observed (Figure 6).

Mutations in the PKD1 gene (encoding Polycistin-1) are accounted for the renal cysts formation [59]. Overexpression of PKD1 in Madin-Darby canine kidney (MDCK) cells has led to decreased apoptosis [60] and silencing of PKD1 has led to an increased apoptosis rate due to a reduced cell adhesion [61]. PKD1 and PKD2 are expressed in a number of tissues and organs, including the ductal epithelial cells in the kidney, liver, pancreas, breast, smooth muscle, endothelial cells of the vasculature and astrocytes in the brain [62]. U87 cell line is an astrocytoma cell line derived from a human malignant glioma and we showed that PKD1 was expressed in this cell line (Figure 6). Consistent with previous studies as well as considering high expression level of PKD1 in most of the brain tumor-derived cell lines, a reduction in PKD1 expression following the overexpression of mir-6165 could justify the increased rate of apoptosis and a change of cell number distribution toward sub-G1 (Figure 4).

DAGLA gene is involved in endocannabinoid pathway shown by mouse knock out model. This pathway refers to a group of neuromodulatory lipids and their receptors [63]. DAGLA gene effect on proliferation is not yet clear to our knowledge. A recent publication has suggested that DAGLA deletion ended in ∼50% less cell proliferation of the hippocampus cells [63]. DAGLA gene has multiple target sites for mir-6165 within its 3′-UTR and it is deducible that overexpression of this miRNA has caused down regulation of this target gene at least at the mRNA level (Figure 6). It is remained to be tested if increased rate of apoptosis is related to the down regulation of DAGLA gene as well.

Co-expression of mRNAs and/or functional similarity of target and host genes of an intronic miRNA have been hypothesized [64]. This hypothesis was tested for NGFR and mir-6165 predicted target genes using GENEMANIA database (Figure S2). There was a co-expression pattern between most of the targets and NGFR host gene while none of the high scored targets show a direct physical interaction with NGFR gene.

It has been suggested that intronic miRNAs tend to target the genes that are functionally similar to their host genes [64]. Using Funsimmat algorithm (funsimmat.bioinf.mpi-inf.mpg.de) high scored target genes of mir-6165 showed functional similarity with NGFR gene as well. Noteworthy, Diana-mirpath and geneset2miRNA online servers showed that most of the first 20 high scored targets of mir-6165 are also targeted by other miRNAs involved in cancers and brain development like mir-608, mir24 and mir-637. How this network of miRNAs with overlapped functions act in cancerous cell and development remain to be tested.

In conclusion, by multiple experimental evidence, our study revealed that mir-6165 is expressed in brain tumor-derived cell lines and primary brain tumor tissues. Overexpression of mir-6165 in U87 cell line increased the cell death rate and down regulated PKD1 and DAGLA target genes, which are involved in apoptosis. Furthermore, there is a strong co-expression network of mir-6165 host and target genes.

## Supporting Information

Figure S1 **Co-expression network of NGFR gene with mir-6165predicted targets.** Although there is a co expression network between targets genes of mir-6165 but none of these targets have direct interaction with NGFR host gene. The targets of mir-6165have been shown by the larger nodes.
(TIF)

Figure S2 **Percent of putative targets of Hsa-mir-6165 in different tissues.** Analysis of mir-6165 putative target genes by DAVID showed more than 50 percent of predicted target genes are expressed in brain related tissues.
(TIF)

## References

[1] Gao X, Daugherty RL, Tourtellotte WG (2007). Regulation of low affinity neurotrophin receptor (NGFR ) by early growth response (egr) transcriptional regulators. Mol. Cell.. Neurosci 36(4),.

[2] Cantarella G, Lempereur L, Presta M, Ribatti D, Lombard G (2002). Nerve growth factor-endothelial cell interaction leads to angiogenesis in vitro and in vivo.. The FASEB J. 16(10),.

[3] Caporali A, Pani E, Horrevoets AJG, Kraenkel N, Oikawa A (2008). Neurotrophin NGFR receptor (NGFR ) promotes endothelial cell apoptosis and inhibits angiogenesis: Implications for diabetes-induced impaired neovascularization in ischemic limb muscles. Circ.. Res 103(2),.

[4] Salis MB, Graiani G, Desortes E, Caldwell RB, Madeddu P (2004). Nerve growth factor supplementation reverses the impairment, induced by type 1 diabetes, of hindlimb post-ischaemic recovery in mice.. Diabetologia 47(6),.

[5] Wang S, Bray P, McCaffrey T, March K, Hempstead BL (2000). p75(NTR) mediates neurotrophin-induced apoptosis of vascular smooth muscle cells. Am.. J. Pathology 157(4),.

[6] Rocha AS, Risberg B, Magalhães J, Trovisco V, de Castro IV (2006). The NGFR neurotrophin receptor is widely expressed in conventional papillary thyroid carcinoma.. Human Pathology 37(5),.

[7] Krygier S, Djakiew D (2001). Molecular characterization of the loss of NGFR expression in human prostate tumor cells. Mol.. Carcinog 31(1),.

[8] Guate JL, Fernández N, Lanzas JM, Escaf S, Vega JA (1999). Expression of p75(LNGFR) and trk neurotrophin receptors in normal and neoplastic human prostate.. BJU International 84(4),.

[9] Marchetti D, Aucoin R, Blust J, Murry B, Greiter-Wilke A (2004). NGFR neurotrophin receptor functions as a survival receptor in brain-metastatic melanoma cells. J. Cell.. Biochem 91(1),.

[10] Kanik AB Yaar M Bhawan J 1996 NGFR nerve growth factor receptor staining helps identify desmoplastic and neurotropic melanoma. Journal of Cutaneous Pathology 23(3), 205–210 DOI:10.1111/j.1600–0560.1996.tb01468.x. 10.1111/j.1600-0560.1996.tb01468.x 8793654

[11] Jin H, Pan Y, Zhao L, Zhai H, Li X (2007). NGFR neurotrophin receptor suppresses the proliferation of human gastric cancer cells.. Neoplasia 9(6),.

[12] Dimaras H, Gallie BL (2008). The NGFR neurotrophin receptor is a tumor suppressor in human and murine retinoblastoma development. Int.. J. Cancer 122(9),.

[13] Stephan H, Zakrzewski JL, Bölöni R, Grasemann C, Lohmann DR (2008). Neurotrophin receptor expression in human primary retinoblastomas and retinoblastoma cell lines.. Pediatric Blood and Cancer 50(2),.

[14] Dimaras H, Coburn B, Pajovic S, Gallie BL (2006). Loss of NGFR neurotrophin receptor expression accompanies malignant progression to human and murine retinoblastoma. Mol.. Carcinog 45(5),.

[15] Johnston AL, Lun X, Rahn JJ, Liacini A, Wang L (2007). The NGFR neurotrophin receptor is a central regulator of glioma invasion.. PLoS Biol 5(8).

[16] Ramos A, Wai CH, Forte S, Dickson K, Boutilier J (2007). Hypo-osmolar stress induces NGFR expression by activating Sp1-dependent transcription.. J. Neurosci 27(6),.

[17] Blöchl A, Blöchl R (2007). A cell-biological model of NGFR signaling.. J. Neurochem 102(2),.

[18] Vilar M, Charalampopoulos I, Kenchappa RS, Reversi A, Klos-Applequist JM, Karaca E (2009). Ligand-independent signaling by disulfide-crosslinked dimers of the p75 neurotrophin receptor.. Journal of Cell Science 122(18),.

[19] Barker PA (2009). A p75NTR pivoting paradigm propels perspicacity.. Neuron 62(1),.

[20] Ambros V (2004). The functions of animal microRNAs.. Nature 431(7006),.

[21] Griffiths-Jones S, Grocock RJ, van Dongen S, Bateman A, Enright AJ (2006). miRBase: MicroRNA sequences, targets and gene nomenclature Nucleic Acids Res 34(Database issue),. http://dx.doi.org/10.1093/nar/gkj112.

[22] Baskerville S, Bartel DP (2005). Microarray profiling of microRNAs reveals frequent coexpression with neighboring miRNAs and host genes.. RNA 11(3),.

[23] Lin S, Miller JD, Ying S (2006). Intronic microRNA (miRNA).. Journal of Biomedicine and Biotechnology.

[24] Musiyenko A, Bitko V, Barik S (2008). Ectopic expression of miR-126*, an intronic product of the vascular endothelial EGF-like 7 gene, regulates prostein translation and invasiveness of prostate cancer LNCaP cells.. Journal of Molecular Medicine 86(3),.

[25] Rodriguez A, Griffiths-Jones S, Ashurst JL, Bradley A (2004). Identification of mammalian microRNA host genes and transcription units.. Genome Res, 14(10 A),.

[26] Lin S, Chang D, Wu D, Ying S (2003). http://dx.doi.org/10.1016/j.bbrc.2003.09.070.

[27] Weber MJ (2005). New human and mouse microRNA genes found by homology search.. FEBS J 272(1),.

[28] Berezikov E, Cuppen E, Plasterk RHA (2006). Approaches to microRNA discovery. Nat. Genet 38(SUPPL.. 1),.

[29] Yoon BJ, Vaidyanathan PP (2007). Computational Identification and Analysis of noncoding RNAs.. IEEE Signal Process 24(1),.

[30] Oulas A, Reczko M, Poirazi P (2009). MicroRNAs and cancer - the search begins! IEEE Transactions on Information Technology in Biomedicine 13(1),. http://dx.doi.org/ 10.1109/TITB.2008.2007086.

[31] Li L, Xu J, Yang D, Tan X, Wang H (2010). Computational approaches for microRNA studies: a review.. Mammalian Genome 21(1–2),.

[32] Oulas A, Boutla A, Gkirtzou K, Reczko M, Kalantidis K (2009). Prediction of novel microRNA genes in cancer-associated genomic regions–a combined computational and experimental approach.. Nucleic Acids Res 37(10),.

[33] Jiang P, Wu H, Wang W, Ma W, Sun X (2007). MiPred: Classification of real and pseudo microRNA precursors using random forest prediction model with combined features.. Nucleic Acids Res 35(Web Server issue),.

[34] Tyagi S, Vaz C, Gupta V, Bhatia R, Maheshwari S (2008). http://dx.doi.org/10.1016/j.bbrc.2008.05.134.

[35] Helvik SA, Snøve JrO, Sætrom P (2007). Reliable prediction of drosha processing sites improves microRNA gene prediction.. Bioinformatics 23(2),.

[36] Ritchie W, Théodule F, Gautheret D (2008). Mireval: A web tool for simple microRNA prediction in genome sequences.. Bioinformatics 24(11),.

[37] Kent WJ (2002). BLAT - the BLAST-like alignment tool.. Genome Res 12(4),.

[38] Maragkakis M, Reczko M, Simossis VA, Alexiou P, Papadopoulos GL (2009). DIANA-microT web server: Elucidating microRNA functions through target prediction. Nucleic Acids Res 37(SUPPL.. 2),.

[39] Maragkakis M, Alexiou P, Papadopoulos GL, Reczko M, Dalamagas T (2009). Accurate microRNA target prediction correlates with protein repression levels.. BMC Bioinformatics10,.

[40] Gkirtzou K, Tsamardinos I, Tsakalides P, Poirazi P (2010). MatureBayes: A probabilistic algorithm for identifying the mature miRNA within novel precursors.. PLoS ONE.

[41] Zhao D, Wang Y, Luo D, Shi X, Wang L (2010). http://dx.doi.org/10.1016/j.artmed.2010.03.004.

[42] Sewer A, Paul N, Landgraf P, Aravin A, Pfeffer S (2005). Identification of clustered microRNAs using an ab initio prediction method.. BMC Bioinformatics 6,.

[43] Knudsen S (1999). Promoter2.0: For the recognition of PolII promoter sequences.. Bioinformatics 15(5),.

[44] Papadopoulos GL, Alexiou P, Maragkakis M, Reczko M, Hatzigeorgiou AG (2009). DIANA-mirPath: Integrating human and mouse microRNAs in pathways.. Bioinformatics 25(15),.

[45] Antonov AV, Dietmann S, Wong P, Lutter D, Mewes HW (2009). GeneSet2miRNA: Finding the signature of cooperative miRNA activities in the gene lists. Nucleic Acids Res 37(SUPPL.. 2),.

[46] Warde-Farley D, Donaldson SL, Comes O, Zuberi K, Badrawi R (2010). The GeneMANIA prediction server: Biological network integration for gene prioritization and predicting gene function. Nucleic Acids Res 38(SUPPL.. 2),.

[47] Dennis G, Sherman BT, Hosack DA, Yang J, Gao W, Lane HC (2003). DAVID: Database for annotation, visualization, and integrated discovery.. Genome Biology.

[48] Andrews P, Damjanov I, Simon D (1984). Pluripotent embryonal carcinoma clones derived from the human teratocarcinoma cell line Tera-2.. Differentiation in vivo and in vitro Lab Invest.

[49] Malakootian M, Mowla SJ, Saberi H, Asadi MH, Atlasi Y (2010). Differential expression of nucleostemin, a stem cell marker, and its variants in different types of brain tumors.. Molecular Carcinogenesis 49(9),.

[50] Maniatis T, Fritsch EF, Sambrook J (1982). Molecular Cloning: A Laboratory Manual..

[51] Mestdagh P, Van Vlierberghe P, De Weer A, Muth D, Westermann F (2009). A novel and universal method for microRNA RT-qPCR data normalization.. Genome Biology.

[52] Lewis BP, Shih I, Jones-Rhoades MW, Bartel DP, Burge CB (2003). Prediction of mammalian MicroRNA targets.. Cell 115(7),.

[53] Bentwich I (2005). Prediction and validation of miRNAs and their targets.. FEBS Lett 579,.

[54] Altuvia Y, Landgraf P, Lithwick G, Elefant N, Pfeffer S (2005). Clustering and conservation patterns of human microRNAs.. Nucleic Acids Res 33(8),.

[55] Lu J, Getz G, Miska EA, Alvarez-Saavedra E, Lamb J (2005). MicroRNA expression profiles classify human cancers.. Nature 435(7043),.

[56] Rhodes DR, Kalyana-Sundaram S, Mahavisno V, Varambally R, Yu J (2007). Oncomine 3.0: Genes, pathways, and networks in a collection of 18,000 cancer gene expression profiles.. Neoplasia 9(2),.

[57] Aranha MM, Santos DM, Xavier JM, Low WC, Steer CJ (2010). Apoptosis-associated microRNAs are modulated in mouse, rat and human neural differentiation.. BMC Genomics.

[58] Cheng AM, Byrom MW, Shelton J, Ford LP (2005). Antisense inhibition of human miRNAs and indications for an involvement of miRNA in cell growth and apoptosis.. Nucleic Acids Research 33(4),.

[59] Hughes J, Ward CJ, Peral B, Aspinwall R, Clark K (1995). The polycystic kidney disease 1 (PKD1) gene encodes a novel protein with multiple cell recognition domains.. Nature Genetics 10,.

[60] Boletta A, Qian F, Onuchic LF, Bhunia AK, Phakdeekitcharoen B (2000). http://dx.doi.org/10.1016/S1097-2765(00)00123-4.

[61] Battini L, Fedorova E, Macip S, Li X, Wilson PD (2006). Stable Knockdown of Polycystin-1 Confers Integrin-α2β1–Mediated Anoikis Resistance. J. Am. Soc.. Nephrol 17,.

[62] Zhou J (2009). Polycystins and Primary Cilia: Primers for Cell Cycle Progression.. Annual Review of Physiology.

[63] Gao Y, Vasilyev DV, Goncalves MB, Howell FV, Hobbs C (2010). Loss of retrograde endocannabinoid signaling and reduced adult neurogenesis in diacylglycerol lipase knock-out mice.. Journal of Neuroscience 30(6),.

[64] Lutter D, Marr C, Krumsiek J, Lang EW, Theis FJ (2010). Intronic microRNAs support their host genes by mediating synergistic and antagonistic regulatory effects.. BMC Genomics.

