# Supplementary figures and images for: Experimental Verification of a Predicted Intronic MicroRNA in Human NGFR Gene with a Potential Pro-Apoptotic Function
Source: PLoS One. 2012 Apr 27;7(4):e35561. doi: 10.1371/journal.pone.0035561 (PMC3338703; doi:10.1371/journal.pone.0035561)

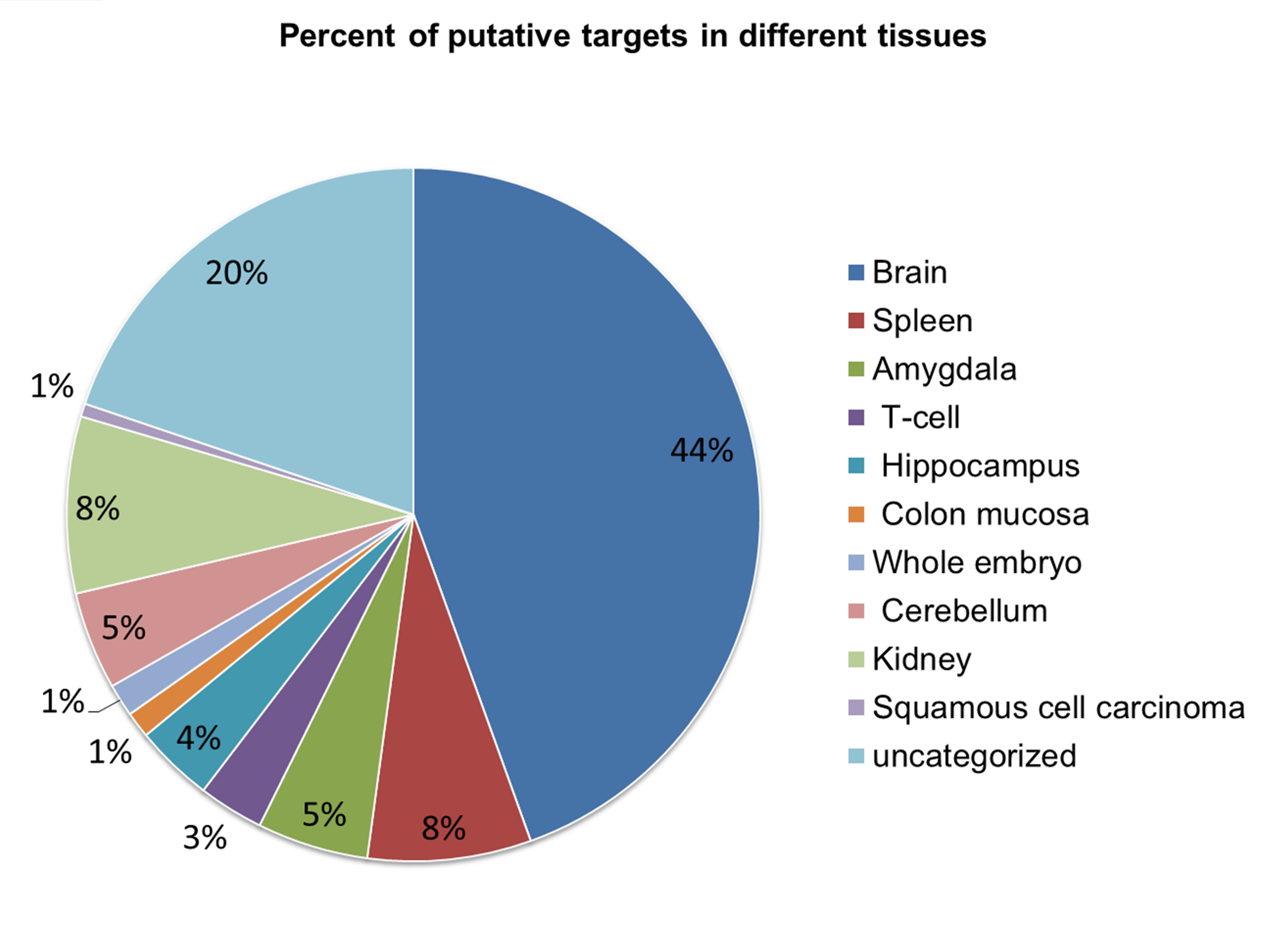

Supplement: Figure S1 — Co-expression network of NGFR gene with mir-6165predicted targets. Although there is a co expression network between targets genes of mir-6165 but none of these targets have direct interaction with NGFR host gene. The targets of mir-6165have been shown by the larger nodes. (TIF) [file pone.0035561.s001.tif]

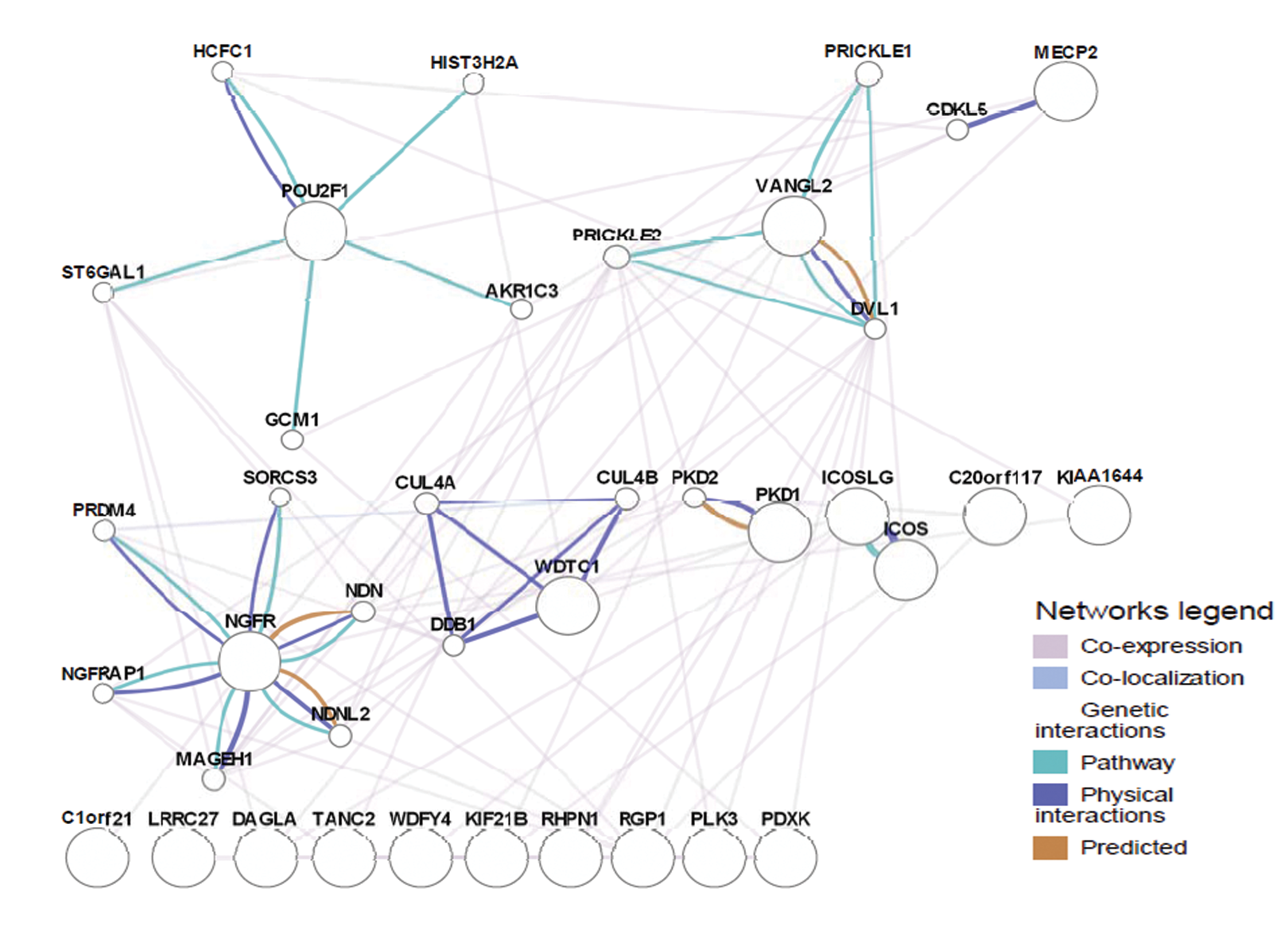

Supplement: Figure S2 — Percent of putative targets of Hsa-mir-6165 in different tissues. Analysis of mir-6165 putative target genes by DAVID showed more than 50 percent of predicted target genes are expressed in brain related tissues. (TIF) [file pone.0035561.s002.tif]
